# Potential of MMP-9 based nanoparticles at optimizing the cow dry period: pulling apart the effects of MMP-9 and nanoparticles

**DOI:** 10.1038/s41598-020-67176-2

**Published:** 2020-07-09

**Authors:** L. Gifre-Renom, J. V. Carratalá, S. Parés, L. Sánchez-García, N. Ferrer-Miralles, A. Villaverde, A. Bach, Elena Garcia-Fruitós, Anna Arís

**Affiliations:** 1 Department of Ruminant Production, Institut de Recerca i Tecnologia Agroalimentàries (IRTA), 08140 Caldes de Montbui, Spain; 2 Institut de Biotecnologia i de Biomedicina, Universitat Autònoma de Barcelona, 08193 Cerdanyola del Vallès, Spain; 3 Departament de Genètica i de Microbiologia, Universitat Autònoma de Barcelona, 08193 Cerdanyola del Vallès, Spain; 4 CIBER de Bioingeniería, Biomateriales y Nanomedicina (CIBER-BBN), 08193 Cerdanyola del Vallès, Spain; 5 Institució Catalana de Recerca i Estudis Avançats (ICREA), Barcelona, Spain

**Keywords:** Disease prevention, Animal biotechnology, Nanobiotechnology

## Abstract

The cow dry period is a non-milking interval where the mammary gland involutes and regenerates to guarantee an optimal milk production in the subsequent lactation. Important bottlenecks such as the high risk of intramammary infections complicate the process. Antibiotics have been routinely used as a preventive treatment but the concerns about potential antibiotic resistance open a new scenario in which alternative strategies have to be developed. Matrix metalloproteinase-9 (MMP-9) is an enzyme able to degrade the extracellular matrix, triggering the involution and immune function of cow mammary gland. We have studied the infusion into the mammary gland of MMP-9 inclusion bodies as protein-based nanoparticles, demonstrating that 1.2 mg of MMP-9 enhanced the involution and immune function of the cow mammary gland. However, the comparison of the effects triggered by the administration of an active and an inactive form of MMP-9 led to conclude that the response observed in the bovine mammary gland was mainly due to the protein format but not to the biological activity of the MMP-9 embedded in the inclusion body. This study provides relevant information on the future use of protein inclusion bodies in cow mammary gland and the role of MMP-9 at dry-off.

## Introduction

The dry period is crucial to optimize milk production in dairy cattle^1^. During this period, the mammary gland regresses and, after that, it proliferates and differentiates to allow optimal milk production in the subsequent lactation. However, the presence of galactopoietic hormones due to a concomitant pregnancy does not facilitate the beginning of involution and delays the activation of the immune system, which orchestrates all this process^2^. Moreover, the high amount of milk accumulated in the mammary gland at dry-off exert high intra-mammary pressure and may lead to milk leakage, which in turn maintains the teat canal opened and full of nutrients, increasing the risk of a pathogen invasion^3^. When activated, the immune system recruits macrophages and neutrophils, which could fight against a possible infection^4^. Yet their phagocytic activity against pathogens is diminished at dry-off, as phagocytes are engaged at engulfing milk fat, cell debris, and other compounds derived from milk and accumulated in the mammary gland^4^. To reduce the risk of mastitis, antibiotics are infused routinely into the mammary gland at dry-off. However, the preventive use of antibiotics has raised concerns about the emergence of antibiotic resistances. In this context, there is a need to find new strategies to boost the immune system of the mammary gland and its involution at dry-off.

Recently, new strategies based on the use of matrix metalloproteinase-9 (MMP-9) have been studied mainly to modulate infiltration of immune cells and involution at dry-off. Matrix metalloproteinase-9 is a tissue-remodelling enzyme that degrades the extracellular matrix (ECM) and, in the mammary gland, is physiologically released by mammary epithelial cells and neutrophils entering into the tissue during the involution process^5,6^. It has been previously demonstrated that the proteolytic degradation of the ECM is a key factor during the loss of differentiation of mammary epithelial cells and the induction of apoptosis and involution^7^. Hence, in a previous work we hypothesized that exogenous administration of a recombinant MMP-9 (rMMP-9), which was not sensible to tissue inhibitors of metalloproteinases 1 and 3 (TIMP1 and TIMP3), could represent a strategy to accelerate tissue involution at dry-off. The administration of rMMP-9 into the mammary gland at dry-off was tested^8^ using two recombinant protein formats: a soluble form, and a nanoparticulated format, also known as inclusion body (IB). Inclusion bodies are protein-based nanoparticles of few hundred nanometers formed during recombinant protein production processes^9^ showing a clear biomedical potential^10^. Parés *et al*. (2017) reported an increase in the local immune response and mammary involution when administering rMMP-9 as an IB at dry-off in dairy cows, whereas the administration of rMMP-9 in a soluble form only increased the endogenous MMPs without affecting general parameters of immune stimulation and mammary involution markers^8^. These findings encouraged us to elucidate whether the observed effects of rMMP-9 IBs in the bovine mammary gland were due to a different performance of rMMP-9 embedded in the nanoparticles compared with a soluble form, or due to the effect of the nanoparticle or IB format itself. For this, we have determined *in vivo*, first, the lowest dose of rMMP-9 IBs that boost innate immunity and mammary involution, and second, whether this was due to the nanoparticle format or to the inherent properties of the rMMP-9 comprised in these IBs^11^. Through the comparison of the effects triggered by the administration of an inactive form of rMMP-9 IB at dry-off with its active counterpart, the real potential of rMMP-9 IBs during the cow dry period has been distinguished.

## Results and Discussion

### Determining the minimal inflammatory dose of rMMP-9 nanoparticles

**Figure 1:**
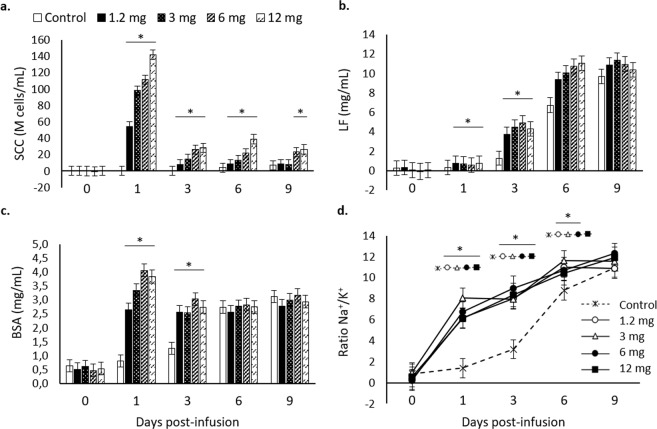
Mammary gland involution markers analysed from mammary gland secretions for the tested doses (0 –control–, 1.2, 3, 6, and 12 mg) of inclusion bodies containing matrix metalloproteinase-9 along 0, 1, 3, 6, and 9 d post-infusion. Non-transformed means and SEM (error bars) are represented while *P*-values were obtained from transformed data, when necessary. Asterisks depict significant differences between treatments and the control group within time. (**a**) Somatic cell counts (SCC) are expressed in million (10^6^) cells per mL (M cells/ml); *P* < 0.0001. (**b**) Lactoferrin (LF); *P* < 0.0001. (**c**) Bovine serum albumin (BSA); *P* < 0.0001. (**d**) Na^+^/K^+^ ratio; *P* < 0.0001. For differences between different treatments within each time point, see Table S1 in the Supplementary Material.

The greatest tested dose (12 mg) of MMP-9 IBs at dry-off was previously reported by Parés *et al*. (2017) as a potent immunostimulator of the bovine mammary gland^8^. Herein, in Exp. 1, three lower doses were evaluated along with the 12-mg dose. All used doses enhanced the recruitment of immune cells into mammary gland (namely SCC) up to 6 d post dry-off (Fig. 1a). At day 9, the myeloid cells recruited in the controls started to rise but were still below those obtained with the 12- and 6-mg doses (Fig. 1a). Lactoferrin synthesis, whose rise is also associated with an augmented immune activity^12^, increased at day 1 after dry-off until day 3 in all the tested doses (Fig. 1b). The involution markers BSA and Na^+^/K^+^, whose increase reflects the disruption of tight junction and the mixture of blood components in milk secretion^13^, followed similar patterns as lactoferrin with a marked increase at day 1 after infusions (Fig. 1c,d). For all doses tested in Exp. 1 there was a shift in the levels of the analysed parameters at day 1 and these values were kept above controls until day 3 or day 6 for BSA and Na^+^/K^+^, respectively (Fig. 1c,d). Importantly, this experiment replicated the results observed by Parés *et al*. (2017)^8^, consolidating the potential of rMMP-9 IBs to locally stimulate the recruitment of immune cells in the mammary gland at dry-off and to accelerate the onset of involution biomarkers after 24 h of infusions.

**Figure 2:**
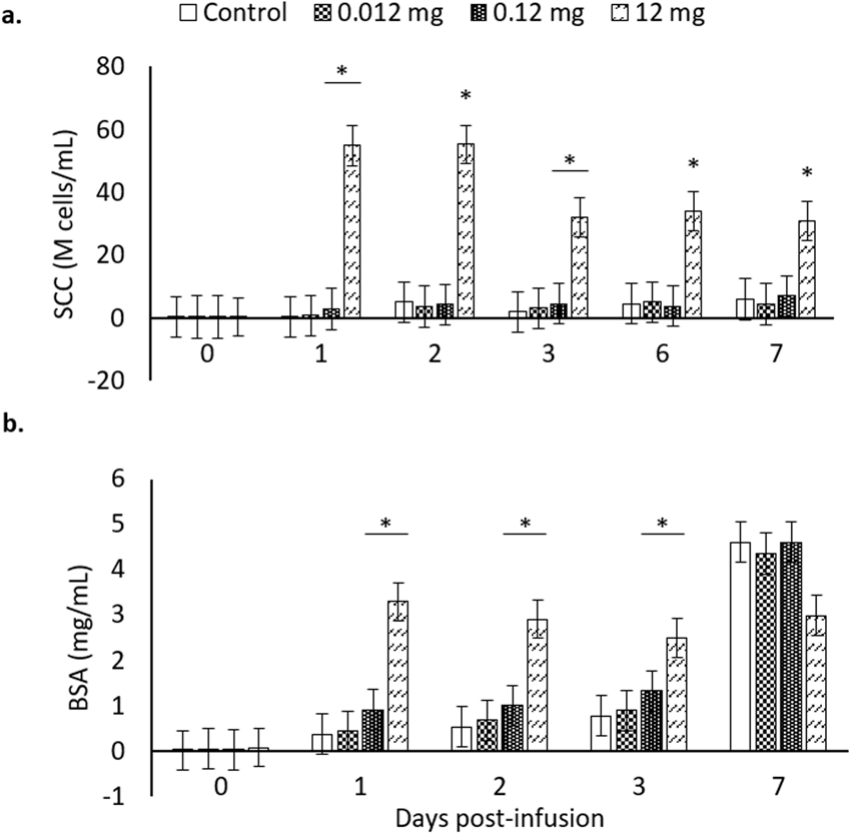
Mammary gland involution markers analysed from mammary gland secretions for the tested doses (0 –control–, 0.012, 0.12, and 12 mg) of inclusion bodies containing matrix metalloproteinase-9 along 0, 1, 2, 3, 6, and 7 d post-infusion. Non-transformed means and SEM (error bars) are represented while *P*-values were obtained from transformed data, when necessary. Asterisks depict significant differences between treatments and the control group within time. (**a**) Somatic cell counts (SCC) are expressed in million (10^6^) cells per mL (M cells/ml); *P* < 0.0001. (**b**) Bovine serum albumin (BSA); *P* < 0.0001. For differences between different treatments within each time point, see Table S2 in the Supplementary Material.

Since all the tested doses in Exp. 1 (Fig. 1) induced similar effects during the week following dry-off, either regarding the immune stimulation or involution parameters, Exp. 2 was conducted to test lower doses of rMMP-9 IBs (Fig. 2). In this case, there was a lack of effect of low doses only observing a slight increase over control quarters with 0.12 mg (Fig. 2). Given that the minimum amount of rMMP-9 IBs eliciting a clear immunostimulating effect in the mammary gland was 1.2 mg (Fig. 2), this dose was chosen to further analyse the effects of rMMP-9 in Exp. 3.

### Differentiation of rMMP-9 and IB format effects

**Figure 3:**
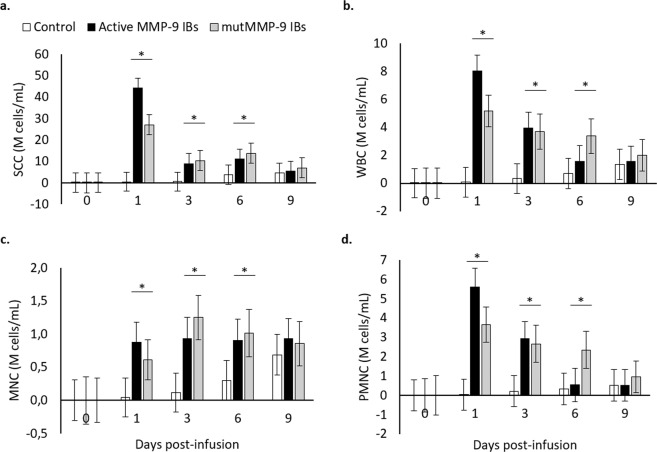
Mammary gland cell populations analysed from mammary gland secretions for the 1.2 mg dose of inclusion bodies containing active (MMP-9 IBs) or inactive (mutMMP-9 IBs) matrix metalloproteinase-9 and control, along 0, 1, 3, 6, and 9 days-post infusion. All cell measurements are expressed in million (10^6^) cells per mL (M cells/ml). Non-transformed means and SEM (error bars) are represented while *P*-values were obtained from transformed data, when necessary. Asterisks depict significant differences between treatments and the control group within time. (**a**) Somatic cell counts (SCC); *P* < 0.0001. (**b**) White blood cells (WBC); *P* < 0.0001. a^t^ *P* = 0.053. (**c**) Mononuclear cells (MNC); *P* < 0.0001. (**d**) Polymorphonuclear cells (PMNC); *P* < 0.0001. For differences between different treatments within each time point, see Table S3 in the Supplementary Material.

Aiming to determine whether the detected immune response triggered in the mammary gland by rMMP-9 IBs^8^ (Fig. 1) was only due to the nanoparticle format or whether the activity of the MMP-9 embedded in such nanoparticles was also relevant, as observed in mice^14^, an Exp. 3 was conducted. We compared the performance between rMMP-9 nanoparticles and the mutant and inactive rMMP-9 counterpart at the established dose of 1.2 mg in bovine mammary gland. Surprisingly, there was no difference between the performance of inactive or active rMMP-9 nanoparticles in the recruitment of immune cells, neither in the general (i.e., WBC) nor in mononuclear or polymorphonuclear cells, being these levels much greater than in control quarters for both treatments (Fig. 3). As expected, the main recruited cells were neutrophils (determined as PMNCs, Fig. 3d), as they are the first immune cell to arrive to the inflammatory site, and in agreement with the behaviour previously observed in mice after the administration of rMMP-9 IBs^14^. However, in the mouse model, the inactive rMMP-9 nanoparticles only induced a slightly and transitory inflammatory effect, whereas the active form had a clear and sustained effect over time^14^. Thus, the performance of mutMMP-9 nanoparticles was different in the mouse intra-dermis model compared with the bovine mammary gland.

**Figure 4:**
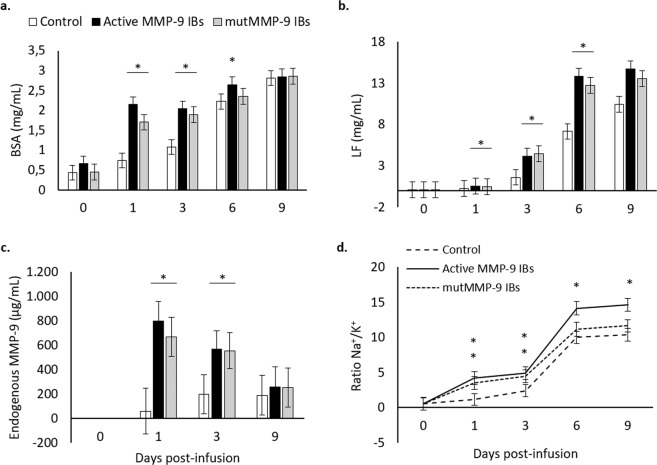
Mammary gland involution markers analysed from mammary gland secretions for the 1.2 mg dose of inclusion bodies containing active (MMP-9 IBs) or inactive (mutMMP-9 IBs) matrix metalloproteinase-9 and control along 0, 1, 3, 6, and 9 d post-infusion. Non-transformed means and SEM (error bars) are represented while *P*-values were obtained from transformed data when necessary. Asterisks depict significant differences between treatments and the control group within time. (**a**) Bovine serum albumin (BSA); *P* < 0.0001. (**b**) Lactoferrin (LF); *P* < 0.0001. (**c**) Endogenous MMP-9 was analysed only for selected times: 0, 1, 3 and 9 d post-infusions; *P* < 0.05. (**d**) Na^+^/K^+^ ratio; *P* < 0.0001. For differences between different treatments within each time point, see Table S4 in the Supplementary Material.

When other immune or involution parameters of bovine mammary gland were assessed, only in very few instances the active rMMP-9 nanoparticles had a slightly different performance compared to the inactive rMMP-9 form. Concretely, BSA levels were greater at days 1 and 6 in quarters treated with active rMMP-9 nanoparticles (Fig. 4a), suggesting that MMP-9 had some activity behind the unspecific format effects. Also, Na^+^/K^+^ ratio increased at day 6 and this was sustained at day 9 (Fig. 4d). This indicates that the splitting time-point for both specific and unspecific effects of rMMP-9 IBs in bovine mammary gland at dry-off may occur later on, compared with the murine model^14^. However, during the analysed time in Exp. 3, and as observed for cellular recruitment, there were no rMMP-9-specific effects in lactoferrin nor in endogenous MMP-9 levels (Fig. 4b,c). Thus, these results confirm that the obtained effects in the bovine mammary gland were mainly due to the format (i.e., IB) but not due to the activity of the protein embedded in these nanoparticles. Again, this was an unexpected outcome because, in mice, the effect of MMP-9 could be clearly differentiated already at day 1 after injections from the inflammatory consequences of the nanoparticle format^14^. The question now is what is causing such different effects between the two animal models? Divergence in the MMP-9 nanoparticle effects observed in mice model and in bovine mammary gland could be explained by important differences between the two *in vivo* models. On one hand, the mice model was knock-out for endogenous MMP-9, favouring a clear split between the MMP-9-specific effect from the format-linked unspecific effect. Nonetheless, in bovine, mammary gland endogenous MMP-9 seems not to have an important role at dry-off^8^. On the other hand, lactating and involuting mammary glands are very immune-active and responsive organs that, in fact, have been compared with strong mucosal immune programs^15^. This agrees with our results indicating that while in mice the nanoparticle unspecific effect was limited to 24 h, in the dairy cow mammary gland this was extended for a minimum of 9 d (except for Na^+^/K^+^ ratio, differenced at day 6, and the short specific effect in BSA). In this context, the same stimulus could trigger a greater inflammatory effect in cows than in rodents. Moreover, it has been demonstrated that soluble rMMP-9 does not exert any effect on immune and involution parameters at the beginning of bovine dry period^8^. This finding indicates that the protein embedded in the nanoparticles is not as relevant as in the mouse air pouches model, specifically designed to evaluate the MMP-9 activity and further infiltration of immune cells.

The effects of the IB format on the inflammation of a host were also previously studied by Torrealba *et al*. (2016a)^16^. They have demonstrated that protein nanoparticles could induce inflammation in Zebrafish and act as an adjuvant^16^, and this effect could be even increased through using nanoparticles composed by proteins with a relevant immune function, such as a cytokine^17^. Similar to results herein, Torrealba *et al*. (2016b) also described a dose-dependent effect when an unspecific protein like GFP was injected in Zebrafish, with a fast-induced immunostimulating effect^17^. This is in agreement with other studies performed previously with LPS^18,19^, chitosan^20^, *Panax ginseng* extracts^21,22^, among others, in which a rapid immunostimulating effect is observed at dry-off.

## Conclusions

Protein nanoparticles or inclusion bodies trigger a clear immunostimulant effect in the bovine mammary gland at dry-off. Matrix metalloproteinase-9 protein forming such nanostructures exerts no relevant effect in the context of the bovine dry-off. Thus, protein nanoparticles could be considered as an appealing strategy at bovine dry-off to accelerate this process and enhance the immune protection although MMP-9 protein itself does not provide any extra value during the first week of the dry period.

## Materials and Methods

### Bacteria strains and plasmids

*Lactococcus lactis* subsp. *cremoris* NZ9000 double mutant ClpP^-^ HtrA^-^ (*clpP-htrA*; Em^R^) strain^23,24^ (kindly provided by INRA (Jouy-en-Josas, France); patent n. EP1141337B1) was used to recombinantly produce the MMP-9 proteins. The genes encoding for an active bovine rMMP-9 fragment (Phe107 - Pro449, NCBI, NM_174744.2) and for the same rMMP-9 fragment with the E402Q single mutation^25,26^, which makes it an inactive rMMP-9 proteoform, were cloned in pNZ8148 plasmid (Cm^R^) and transformed into competent *L. lactis clpP-htrA* cells. Both genes were fused to a His-tag in the C-terminal and were codon-optimised (Geneart) for *L. lactis*^11^.

### Protein Production in *L. lactis*

Both, active and inactive rMMP-9 were produced in *L. lactis*, which was grown under static conditions at 30 °C in M17 broth supplemented with 0.5% glucose, 5 µg/mL chloramphenicol and 2.5 µg/mL erythromycin. Cultures were re-inoculated to an initial OD_600nm_ of 0.05 and protein expression was induced with 12.5 ng/mL nisin when the OD_600nm_ reached values between 0.4 and 0.6. The recombinant proteins were produced along 3 h and bacteria were recovered by centrifugation at 6,000 *x g* for 30 min at 4 °C and stored at −80 °C until use.

### Purification of IBs

Pellets from 50 mL culture were suspended in 30 mL PBS, frozen/thawed at −80 °C and disrupted with 3 French press (Thermo FA-078A) rounds at 1,500 psi, ice-coated, and with protease inhibitors (cOmplete protease inhibitor cocktail EDTA-free, Roche). After that a previously established purification protocol was applied as described^27^. Samples were tested for sterility by plating an aliquot in agar-M17 plates with 0.5% glucose and incubating them overnight at 30 °C. Aliquots of rMMP-9 IBs and mutant inactive rMMP-9 IBs were tested for purity through SDS-PAGE electrophoresis and Coomassie blue staining (75.7% purity obtained for MMP-9 IBs and 70.3% for mutMMP-9 IBs), and were quantified by interpolation with the bands obtained by a solubilized rMMP-9 as the standard^28^, using ImageJ software (version 1.46r, U. S. National Institutes of Health, Bethesda, Maryland, USA). The activity of the MMP-9 IBs and the inactivity of the mutMMP-9 IBs, were validated *in vitro* both by zymography and DQgelatin (data not shown).

### Protein infusions in cow mammary glands

Thirty-three multiparous pregnant Holstein cows in their second gestation, after 274 to 379 days in lactation and between 215 and 225 days of gestation, producing from 25.5 to 43.7 kg of milk per day during the last week before dry-off, and with <200,000 somatic cell counts (SCC)/mL in milk at dry-off per cow, were selected in this study. Cows were dried abruptly without any previous dietary nor milking routine intervention.

Experiments were performed in accordance with relevant guidelines and regulations under the evaluation and permission of the Ethical Committee of IRTA, protocol number 9705.

Three experiments were performed (Exp. 1, Exp. 2, and Exp. 3); 2 experiments aimed at optimizing the minimum effective protein dose to trigger an immune reaction in the mammary gland at dry off (Exp. 1 and Exp. 2), and the third aimed at dissecting the effects triggered either by the protein activity or by the protein format (Exp. 3).

Udder quarter was used as an independent experimental unit and these were randomly allocated to treatments considering cows a random effect. A total of 46, 44 and 30 quarters used for Exp. 1, Exp. 2 and Exp. 3, respectively (2 animals were discarded due to mastitis and abortion, one for each Exp. 1 and Exp. 2). After the last milking before dry-off and just before protein infusions, 10 mL of mammary gland secretions (MGS) were collected from all quarters as a day 0 sampling.

In the first experiment (Exp. 1), a range of different doses of rMMP-9 IBs were infused into 10 quarters per treatment (due to mastitis, 1 quarter per treatment was discarded, except for control) through the teat canal using sterile blunt tip cannulas immediately after MGS collection at day 0. Namely, 1.2, 3, 6, and 12 mg of rMMP-9 IBs suspended in 10 mL saline solution were infused, and 10 mL saline solution infusions was used as negative controls. Following infusions, all quarters were treated with broad-spectrum antibiotics (Mamyzin secado, Boehringer Ingelheim) following common production practices. At days 1, 3, 6, and 9 after protein infusions, 10 mL of MGS were collected from each quarter. After the last sampling, all glands were sealed with teat sealant. All MGS were analysed fresh for SCC (n = 9 observations) as described below, and aliquots were stored at −80 °C until these were analysed for bovine serum albumin (BSA; n = 9), lactoferrin (LF; n = 8) and Na^+^/K^+^ (n = 9) levels.

The second experiment (Exp. 2) aimed at testing lower doses of rMMP-9. After collecting MGS at day 0, doses of 0.012, 0.12, and 12 mg of MMP-9 IBs and 10 mL of saline solution were infused into 11 quarters for each dose, and all of them were treated with antibiotics as described for Exp. 1. At days 1, 2, 3, 6, and 7 after infusions, MGS were obtained from each quarter and after the last collection, teat sealant was applied. The MGS were analysed fresh for SCC (n = 11), and BSA was analysed from aliquots after preservation at −80 °C (n = 11).

In the third experiment (Exp. 3), the minimum effective dose determined from Exp. 1 and Exp. 2 was used to compare the effects of the active and the inactive rMMP-9 IBs on the mammary gland at dry-off. Thus, rMMP-9 IBs and inactive rMMP-9 IBs (**mutMMP-9** IBs) at selected dose, and 10 mL of saline solution were infused into 10 quarters per treatment and then quarters were treated with antibiotics following previously detailed steps in Exp. 1. At days 1, 3, 6, and 9, after protein infusions, MGS were obtained and analysed fresh for SCC (n = 10) and for immune cell populations (n = 10), and aliquots were stored at −80 °C until analysed for BSA (n = 10), LF (n = 8), Na^+^/K^+^ (n = 10) and endogenous MMP-9 (n = 6).

### Mammary secretion analyses

#### Somatic cell counts

Half mL of each MGS was mixed with half mL Dulbecco’s phosphate-buffered saline (DPBS; GIBCO), inverted several times and centrifuged at 1,000 x g for 2 min at RT. Fat, located on top of the sample mixture, was removed by gently swirling a cotton swab around the top of the centrifuge tube. The supernatants were discarded without touching the cell pellets, and these were suspended in 1 mL DPBS and centrifuged again. Cells were washed twice, repeating the previous steps and were suspended in 0.5 mL DPBS. Cell suspensions were counted using a Scepter 2.0 Handheld Automated Cell Counter (Merck Millipore, Billerica, MA). Cells were diluted when appropriate to obtain a best resolution in the Scepter histograms, and particle counts with diameters below 6 µm were discarded for all samples.

#### Immune cell populations

After SCC, cell suspensions were stored overnight at 4 °C, and sent to the Veterinary Clinic Haematology Service at the Autonomous University of Barcelona (UAB, Barcelona, Spain) for the analyses of immune cell populations. Using a XN-1000 analyser (Sysmex), white blood cells (WBC), polymorphonuclear cells (PMNC) and mononuclear cells (MNC) were differentiated and counted following morphological measurements by flow cytometry, selecting the body fluid mode.

#### Bovine serum albumin quantification

Bovine Serum Albumin (BSA) in MGS was quantified as described elsewhere^29^. One mL of each MGS was centrifuged at 1,000 x g for 10 min at RT and fat was removed with a swab as detailed in the section for SCC determination. A commercial BSA was used as the standard curve and an eight-point serial dilution curve from 60 mg/mL was prepared. Two hundred µL of each supernatant were mixed in 450 µL dH_2_0 and 450 µL of Bromocresol Green working solution (consisting in three parts succinic acid at pH 4 and a part of bromocresol green sodium salt dissolved in 5 mM NaOH, and 0.8% Brij L23). The solutions for all samples and standards were centrifuged at 1,900 x g for 10 min at RT and 150 µL of each supernatant were plated by duplicate in flat bottom transparent 96-well plates and were read at 640 nm. Concentration of BSA in MGSs was interpolated to the 4-parametric standard curve.

#### Lactoferrin quantification

Whole MGS were analysed for lactoferrin concentrations using a bovine lactoferrin ELISA quantitation set (Bethyl Laboratories Inc., Montgomery, TX, USA). A commercial bovine lactoferrin calibrator was used as the standard curve, ranging from 500 to 7.8 ng/ml. After coating the plates with 0.01 mg/mL anti-bovine lactoferrin, followed by a blocking step as indicated in the manufacturer protocol, samples diluted at 1/10,000 or 1/100,000 and standards were plated per duplicate and incubated for 1 h at RT. After several washes, HRP conjugated anti-bovine lactoferrin detection antibody was added at a final concentration of 0.01 µg/mL and incubated for 1 h at RT. Following several washes, 3,3′,5,5′-Tetramethylbenzidine (TMB) substrate solution was added and reaction was stopped after 15 min with 0.18 M H_2_SO_4_. Plates were read at 450 nm and lactoferrin concentrations were interpolated from the 4-parametric standard curve.

#### Sodium and potassium quantification

All samples were analysed for Na^+^ and K^+^ concentrations at the Chemical Analysis Service at the UAB. An aliquot of 0.1 g of MGSs was diluted in Triton X-100 0.1% (v/v). Clotted samples were previously digested in HNO_3_ concentrate in a Ultrawave microwave digestion system (Milestone). Na^+^ and K^+^ levels were determined by inductively coupled plasma-Optical emission spectrometry (ICP-OES; Optima 4300DV, Perkin-Elmer, Waltham, MA, USA).

#### Endogenous MMP-9 zymography

Skimmed MGS were quantified for endogenous MMP-9 activity through zymography analysis. Solubilized MMP-9^28^ was used to prepare an eight-point standard curve ranging from 400 to 25 ng. Sample supernatants were diluted 1/10 and diluted samples and standards were loaded with non-denaturing loading buffer into 10% SDS-PAGE gels containing 1% porcine gelatine. Electrophoresis was run at constant 50 mA and gels were washed twice in 2.5% Triton X-100, once in distilled water, and were incubated in static overnight at 37 °C in developing buffer containing 50 mM Tris pH 7.5, 200 mM NaCl and 10 mM CaCl_2_. Afterwards, Coomassie Blue staining was used to dye the gels for 2 h at RT and these were distained in a methanol-acetic solution. Degradation bands were analysed using ImageJ software and MMP-9 activity quantification was interpolated from the solubilized MMP-9 standard curve.

### Statistical analyses

A total of 46, 44, and 30 quarters were used in Exp. 1, Exp. 2 and Exp. 3, respectively. Quarters were randomly allocated to treatments, considering cows a random effect. For immune cell populations, outliers considered as 2-times standard deviation were discarded. Variables were log-transformed in Exp. 1, Exp. 2 and Exp. 3, or root-transformed for Na^+^/K^+^ in Exp. 1, to normalize data when necessary. Data were analysed using a fixed-effects model using SAS 9.4 (SAS Inst. Inc., Cary, NC). Time, dose and the interaction between time and dose were included the model, with quarters nested within cows and cows as random effects. Time was included as a repeated measure and for each analysed variable, quarters within cows (the error term) was subjected to 2 variance-covariance structures: compound symmetry and autoregressive order 1. The covariance structure that yielded the smallest Schwarz’s Bayesian information criterion was considered the most desirable analysis. Means and standard deviations represented in graphs correspond to non-transformed or back-transformed data, while *P*-values and asterisks correspond to the output from transformed data when required. Additional information on the differences among treatments within each time point can be found in the Supplementary Material.

## Supplementary information

Supplementary information.
