# Supplementary material for: Potential of MMP-9 based nanoparticles at optimizing the cow dry period: pulling apart the effects of MMP-9 and nanoparticles
Source: Sci Rep. 2020 Jul 9;10:11299. doi: 10.1038/s41598-020-67176-2 (PMC7347913; doi:10.1038/s41598-020-67176-2)
Supplement: Supplementary file 1 — Supplementary information. [file 41598_2020_67176_MOESM1_ESM.docx]

Supplementary material

**Potential of MMP-9 based nanoparticles at optimizing the cow dry period: pulling apart the effects of MMP-9 and nanoparticles**

L. Gifre-Renom^1^, J. V. Carratalá^2^, S. Parés^1^, L. Sanchez-Garcia^2^, N. Ferrer-Miralles^2,3,4^, A. Villaverde^2,3,4^, A. Bach^1,5^, Elena Garcia-Fruitós^1^*, Anna Arís^1^*

^1^ Department of Ruminant Production, Institut de Recerca i Tecnologia Agroalimentàries (IRTA), 08140 Caldes de Montbui, Spain.

^2^ Institut de Biotecnologia i de Biomedicina, Universitat Autònoma de Barcelona, 08193 Cerdanyola del Vallès, Spain

^3^ Departament de Genètica i de Microbiologia, Universitat Autònoma de Barcelona, 08193 Cerdanyola del Vallès, Spain

^4^ CIBER de Bioingeniería, Biomateriales y Nanomedicina (CIBER-BBN), 08193 Cerdanyola del Vallès, Spain

^5^ Institució Catalana de Recerca i Estudis Avançats (ICREA), Barcelona, Spain

* Co-corresponding authors: [anna.aris@irta.cat](mailto:anna.aris@irta.cat), [elena.garcia@irta.cat](mailto:elena.garcia@irta.cat)

**Table S1.** Detailed output for the statistical analyses in Figure 1 in the main manuscript. Different letters depict differences between different treatments within each time point.

|  | SCC | | | | | BSA | | | | | LF | | | | | Na^+^/K^+^ | | | | |
| --- | --- | --- | --- | --- | --- | --- | --- | --- | --- | --- | --- | --- | --- | --- | --- | --- | --- | --- | --- | --- |
|  | Days after infusion | | | | | Days after infusion | | | | | Days after infusion | | | | | Days after infusion | | | | |
|  | 0 | 1 | 3 | 6 | 9 | 0 | 1 | 3 | 6 | 9 | 0 | 1 | 3 | 6 | 9 | 0 | 1 | 3 | 6 | 9 |
| Control | a | c | d | d | b | a | c | b | a | a | a | b | b | a | a | a | c | b | b | a |
| 1.2 mg | a | b | c | c | b | a | b | a | a | a | a | a | a | a | a | a | b | a | a | a |
| 3 mg | a | a,b | b,c | b,c | b | a | a | a | a | a | a | a | a | a | a | a | a | a | a | a |
| 6 mg | a | a | a,b | a,b | a | a | a | a | a | a | a | a | a | a | a | a | a,b | a | a | a |
| 12 mg | a | a | a | a | a | a | a | a | a | a | a | a | a | a | a | a | a,b | a | a,b | a |

**Table S2.** Detailed output for the statistical analyses in Figure 2 in the main manuscript. Different letters depict differences between different treatments within each time point.

|  | SCC | | | | | | BSA | | | | |
| --- | --- | --- | --- | --- | --- | --- | --- | --- | --- | --- | --- |
|  | Days after infusion | | | | | | Days after infusion | | | | |
|  | 0 | 1 | 2 | 3 | 6 | 7 | 0 | 1 | 2 | 3 | 7 |
| Control | a | c | b | c | b | b | a | c | c | c | a |
| 0.012 mg | a | c | b | b,c | b | b | a | c | b,c | b,c | a |
| 0.12 mg | a | b | b | b | b | b | a | b | b | b | a |
| 12 mg | a | a | a | a | a | a | a | a | a | a | a |

**Table S3.** Detailed output for the statistical analyses in Figure 3 in the main manuscript. Different letters depict differences between different treatments within each time point.

|  | SCC | | | | | WBC | | | | | MNC | | | | | PMNC | | | | |
| --- | --- | --- | --- | --- | --- | --- | --- | --- | --- | --- | --- | --- | --- | --- | --- | --- | --- | --- | --- | --- |
|  | Days after infusion | | | | | Days after infusion | | | | | Days after infusion | | | | | Days after infusion | | | | |
|  | 0 | 1 | 3 | 6 | 9 | 0 | 1 | 3 | 6 | 9 | 0 | 1 | 3 | 6 | 9 | 0 | 1 | 3 | 6 | 9 |
| Control | a | b | b | b | a | a | b | b | b | a | a | b | b | b | a | a | b | b | c | a |
| Active MMP-9 IBs | a | a | a | a | a | a | a | a | a^t^ | a | a | a | a | a | a | a | a | a | b | a |
| mutMMP-9 IBs | a | a | a | a | a | a | a | a | a | a | a | a | a | a | a | a | a | a | a | a |

a^t^, indicates a tendency to differ (*p*-value = 0.0534, for Active MMP-9 IBs *vs.* Control at day 6 after infusions)

**Table S4.** Detailed output for the statistical analyses in Figure 4 in the main manuscript. Different letters depict differences between different treatments within each time point.

|  | BSA | | | | | LF | | | | | Endogenous MMP-9 | | | | Na^+^/K^+^ | | | | |
| --- | --- | --- | --- | --- | --- | --- | --- | --- | --- | --- | --- | --- | --- | --- | --- | --- | --- | --- | --- |
|  | Days after infusion | | | | | Days after infusion | | | | | Days after infusion | | | | Days after infusion | | | | |
|  | 0 | 1 | 3 | 6 | 9 | 0 | 1 | 3 | 6 | 9 | 0 | 1 | 3 | 9 | 0 | 1 | 3 | 6 | 9 |
| Control | a | c | b | b | a | a | b | b | b | a | . | b | b | a | a | b | b | b | b |
| Active MMP-9 IBs | a | a | a | a | a | a | a | a | a | a | . | a | a | a | a | a | a | a | a |
| mutMMP-9 IBs | a | b | a | b | a | a | a | a | a | a | . | a | a | a | a | a | a | b | b |
